# Surgical hip dislocation in treatment of slipped capital femoral epiphysis

**DOI:** 10.1051/sicotj/2016047

**Published:** 2017-02-10

**Authors:** Mohammed Elmarghany, Tarek M. Abd El-Ghaffar, Mahmoud Seddik, Ahmed Akar, Yousef Gad, Eissa Ragheb, Alessandro Aprato, Alessandro Massè

**Affiliations:** 1 Orthopedic Department, Alazhar University Hospitals Cairo 11675 Egypt; 2 Turin University Hospitals “Centro Traumatologico Ortopedico Hospital” 10126 Turin Italy; 3 San Luigi hospital of Orbassano 10043 Turin Italy

**Keywords:** SCFE, Hip, Surgical hip dislocation

## Abstract

*Background*: Most surgeons advocate in situ fixation of the slipped epiphysis with acceptance of any persistent deformity in the proximal femur [Aronsson DD, Loder RT, Breur GJ, Weinstein SL (2006) Slipped capital femoral epiphysis: current concepts. J Am Acad Orthop Surg 14, 666–679]. This residual deformity can lead to osteoarthritis due to femoroacetabular cam impingement (FAI) [Leunig M, Slongo T, Ganz R (2008) Subcapital realignment in slipped capital femoral epiphysis: surgical hip dislocation and trimming of the stable trochanter to protect the perfusion of the epiphysis. Instr Course Lect 57, 499–507].

*Objective*: The primary aim of our study was to report the results of the technique of capital realignment with Ganz surgical hip dislocation and its reproducibility to restore hip anatomy and function.

*Patients and methods:* This prospective case series study included 30 patients (32 hips, 13 left (Lt) hips, 19 right (Rt) hips) with stable chronic slipped capital femoral epiphysis (SCFE) after surgical correction with a modified Dunn procedure. This study included 22 males and eight females. The mean age of our patients was 14 years (10–18 years). The mean follow-up period was 14.5 months (6–36 months).

*Results:* Thirty hips had excellent and good clinical and radiographic outcomes with respect to hip function and radiographic parameters. Two patients had fair to poor clinical outcome including three patients who developed Avascular Necrosis (AVN). The difference between those who developed AVN and those who did not develop AVN was statistically significant in postoperative clinical scores (*p* = 0.0000). The mean slip angle of the femoral head was 52.5° ± 14.6 preoperatively and was corrected to a mean value of 5.6° ± 8.2° with mean correction of 46.85° ± 14.9° (*p* = 0.0000). The mean postoperative alpha angle was 51.15° ± 4.2° with mean correction of 46.70 ± 14.20 (*p* = 0.0000). In our series, the mean postoperative Harris hip score (HHS) was (96.16 ± 9.7) and the mean improvement was (29.6 ± 9.6) (*p* = 0.0000).

*Conclusions*: The modified Dunn procedure allows to restore the normal proximal femoral anatomy by complete correction of the slip angle. This technique may reduce the probability of secondary osteoarthritis and femoroacetabular cam impingement.

## Introduction

Slipped capital femoral epiphysis (SCFE) is a well-known disorder of the hip in adolescents that is characterized by translation of the upper femoral epiphysis from the metaphysis through the physis [1]. Ernst Müller in 1888 was the first to describe this condition. An incidence from 0.2 (Japan) to 10 (United States) per 100,000 was reported [2]. The term slipped capital femoral epiphysis is a misnomer as the epiphysis is held in the acetabulum by the ligamentum teres, and thus it is actually the metaphysis that moves upward and outward while the epiphysis remains in the acetabulum [1]. In most patients, there is an apparent varus relationship between the head and the neck, but occasionally the slip is into a valgus position, with the epiphysis displaced superiorly in relation to the neck [2]. The failure develops through the growth plate, creating a three-dimensional deformity, with the distal fragment in varus in the coronal plane, in extension in the sagittal plane, and in external rotation in the axial plane [2].

In the vast majority of cases, the etiology is unknown. Multiple theories have been proposed for the etiology of idiopathic SCFE, and it is likely a result of both biomechanical and biochemical factors [3], the combination of these factors resulting in a weakened physis with subsequent failure [4]. The SCFE may be associated with a known endocrine disorder [5], renal failure osteodystrophy [6], or with previous radiation therapy [7], these factors may also be at high risk for bilateralism. Some studies have shown that parathyroid hormone (PTH) and 1, 25-dihydroxyvitamin D [1, 25-(OH)^2^ D] are involved in growth-plate chondrogenesis and matrix mineralization. Levels of 1, 25-(OH)^2^ D were also significantly lower in patients with SCFE. The deficiency of M-PTH or 1, 25-(OH)^2^ D during the growth spurt could result in SCFE [6]. Slipped capital femoral epiphysis is classified according to both the clinical nature and the magnitude of the disorder [7]. The traditional clinical classification of Fahey et al. [8] includes pre-slip, acute, chronic, and acute-on-chronic. The preferred clinical classification system for SCFE is the Loder [9] classification which classifies patients into stable and unstable on the basis of the patient’s ability to bear weight.

Two radiographic classification systems are used. The Wilson [10] classification is based on relative displacement of the epiphysis on the metaphysis. The other classification described by Southwick [11] measures epiphyseal-shaft angle (slip angle).

In slipped capital femoral epiphyses (SCFE), the severity of slippage correlates with poor long-term clinical outcome scores and radiographic evidence of osteoarthritis [3]. In situ fixation of higher-grade SCFE has a low surgical risk [12] and has been advocated by authors who believe the deformed hip has the potential to remodel with some restoration of the disturbed anatomic axes [13, 14]; however, the remodeling potential remains controversial [15].

Despite remodeling, the head-neck offset will remain abnormal [16]. This is the cause of potential impingement of the femoral neck with the acetabular cartilage [17]. Impingement in SCFE has been associated with damage of the acetabular cartilage, which may explain the early onset of osteoarthritis after SCFE [18]. The additional complication with SCFE is the relatively high incidence of Avascular Necrosis (AVN), a devastating complication leading to significant disability in these young patients [19].

Slipped capital femoral epiphysis leads to early osteoarthritis resulting from FAI. Impingement in SCFE has been associated with damage of the acetabular cartilage, which may explain early onset of osteoarthritis after SCFE. In slipped capital femoral epiphysis (SCFE), severity of slippage correlates with poor long-term clinical outcome [18].

Realignment procedures for the treatment of SCFE are subcapital, basicervical, intertrochanteric, and subtrochanteric levels. Osteonecrosis as a complication of the surgery is rare in stable SCFE pinned in situ. The risk of necrosis has been described as almost reciprocally proportional to the distance of correction from the physis, a phenomenon that can be explained by the vulnerability of the blood supply to the epiphysis [19].

The realignment procedures at the level of the deformity (i.e., subcapital level) can result in anatomic or near-anatomic restoration of the proximal femur. As such, it is believed they offer the best chance of correcting the anatomic deformities that can lead to early osteoarthritis [20]. To reduce the risk of osteonecrosis of the epiphysis during capital reorientation, tension of the posterosuperior retinaculum, containing the end branches of the medial femoral circumflex artery, is markedly reduced by wedge resection of varying size and location [21].

The surgical hip dislocation technique, which was originally described by Ganz, allows restoration of normal anatomy of proximal femur with complete correction of the slip angle, such that the probability of secondary osteoarthritis and cam-type FAI may be minimized. It also allows direct inspection and preservation of physeal blood supply [22].

Any child with an SCFE and open physis needs treatment; without stabilization, progression is inevitable, so once a slipped capital femoral epiphysis has been diagnosed, treatment is indicated to prevent progression of the slip [15].

The introduction of a safe method to surgically dislocate the hip has been proposed and applied to the SCFE patient to improve on the current treatment strategies to prevent Femoro-Acetabular Impingement (FAI). In comparison to the cuneiform osteotomy, which requires substantial femoral neck shortening to ensure tension-free correction of the femoral epiphysis, the modified Dunn osteotomy allows safe reduction by removal of the posterior callus and thinning of the femoral neck and hence should minimize leg length differences [23].

The hip joint can be surgically dislocated using other approaches; however, the Ganz method of surgical hip dislocation has several advantages. As the abductor is detached by trochanteric flip osteotomy, rigid fixation of this flip fragment by screws restores immediate stability and allows for early mobilization of the patient. Direct inspection and preservation of physeal blood supply and inspection of intra-articular pathology can be evaluated and treated at the same time [24].

## Materials and methods

From November 2013 to November 2016 a prospective case series study was undertaken at Al-Azhar university hospitals (Al-Hussein and Sayed Galal hospitals), Cairo, Egypt and in Torino university hospitals (Centro Traumatologico Ortopedico or Center for Trauma and Orthopedics (CTO) Hospital and san luigi regione gonzole hospital), Torino, Italy on patients with stable slipped capital femoral epiphysis treated with modified Dunn procedure. Nine patients were treated in Egypt, while 23 patients were treated in Italy. Patients with established necrosis before the procedure or with other medical conditions such as renal insufficiency and unstable slippage or acute traumatic SCFE were excluded from our study.

This prospective case series study included 30 patients (32 hips, 13 Lt hips, 19 Rt hips) with stable chronic slipped capital femoral epiphysis after surgical correction with a modified Dunn procedure. This study included 22 males and eight females. The mean age of our patients was 14 years (10–18 years). The mean duration of symptoms before the operation was 4.56 ± 2.5 months. The mean follow-up period was 14.5 months (6–36 months). The mean preoperative alpha angle was 97.85° ± 13. The mean preoperative slip angle was 52.5° ± 14.6. The Harris hip score was evaluated for all patients preoperatively and its mean was 67 ± 9.3, the mean WOMAC score was 88.4 ± 3.8, the mean Merle d’Aubigne score was 12 ± 1 (Tables 1 and 2).

**Table 1.** Demographics and preoperative slipped capital femoral epiphyses (SCFE) features.

Patient number	Age	Sex	Side	Duration of symptoms (months)	Max follow up (months)	BMI (kg/m^2^)
1	12	M	Rt	7	36	22
2	15	M	Lt	6	35	32
3	18	M	Lt	8	35	25
4	15	M	Rt	8	34	32
5	14	M	Rt	10	32	21
6	13	M	Lt	8	30	26
7	12	M	Bilateral	5	30	30
8				8	27	30
9	14	M	Rt	6	18	30
10	15	M	Rt	3	16	33
11	16	M	Lt	8	20	32
12	14	F	Lt	2	15	24
13	16	M	Rt	2	13	28
14	14	M	Bilateral	3	24	30
15				6	12	30
16	15	M	Lt	3	9	34
17	14	F	Lt	2	15	31
18	13	F	Lt	5	12	27
19	13	F	Rt	6	14	22
20	15	M	Rt	2	14	28
21	14	M	Rt	4	15	29
22	15	M	Rt	5	13	30
23	12	F	Rt	1	12	34
24	13	F	Rt	1	6	30
25	11	M	Lt	5	8	26
26	10	M	Rt	2	10	31
27	11	F	Rt	2	8	30
28	12	M	Lt	3	7	28
29	13	M	Rt	2	7	29
30	13	M	Lt	6	13	27
31	10	F	Rt	2	6	25
32	16	M	Rt	5	6	33

**Table 2.** Preoperative features of slipped capital femoral epiphyses (SCFE) patients.

Patient number	LLD (cm)	Flexion	IR + flexion	ER + flexion	HHS score	WOMAC score	Merle score	Slip angle	Alpha angle
1	3	50	Lost (No IR)	40	68	88	11	70	105
2	2	40		50	70	90	13	52	95
3	2	60		50	66	93	11	58	100
4	2	50		50	64	89	11	38	95
5	3	70		60	62	80	11	68	105
6	1	50		40	72	85	13	32	90
7	1	40		50	60	92	11	26	85
8	1	40		50	60	92	11	23	80
9	2	50		50	70	94	11	64	75
10	2	40		35	66	88	12	43	90
11	2	50		60	67	90	11	57	105
12	1	60		70	68	89	12	62	100
13	1	65		65	69	86	12	62	93
14	2	40		60	73	92	13	58	91
15	1	60		60	73	90	13	38	75
16	1	55		70	68	93	14	60.5	112.7
17	2	60		60	68	82	13	66	105
18	2	40		60	65	85	11	56	100
19	1	40		60	65	86	12	82.1	111.5
20	1	40		70	70	80	13	38	95
21	1	50		70	71	84	11	66	105
22	2	55		65	62	87	12	40.1	112
23	1	45		50	64	83	14	53	95
24	1	70		50	65	91	14	30.5	94.7
25	2	60		60	70	92	11	73.4	146.3
26	1	50		70	69	93	13	50	95
27	1	50		60	72	90	12	53	95
28	2	50		55	64	89	12	58	100
29	1	50		50	63	88	11	39	92
30	2	40		65	61	90	11	62	86
31	2	40		60	62	92	11	61	105
32	2	40		70	62	88	13	45	90

Measurements of slip and alpha angles were done using simple goniometer, TM Reception (High-End)^TM^ Viewer version 4.4, and SYNCHROMED FUJIFILM programs.

### Surgical technique

All operations were performed according to the technique described by Ganz et al. [22], under general anesthesia. The patient was placed in a lateral decubitus position with the leg draped so that it was fully moveable. Antibiotic prophylaxis was administered preoperatively.

**Figure 1.**
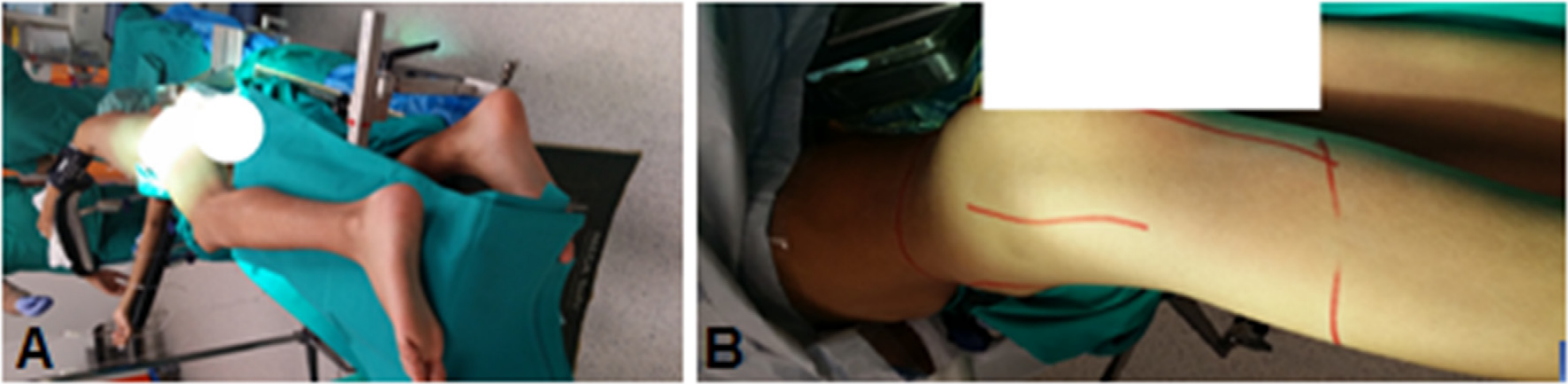
Patient position (A) and skin incision (B) in the Lt hip in patient No. 25.

*A longitudinal lateral incision* (Figure 1) was made centered over the greater trochanter with a sharp dissection carried down to the fascia lata, the approach carried out through the Gibson interval between tensor fascia lata and gluteus maximus.

**Figure 2.**
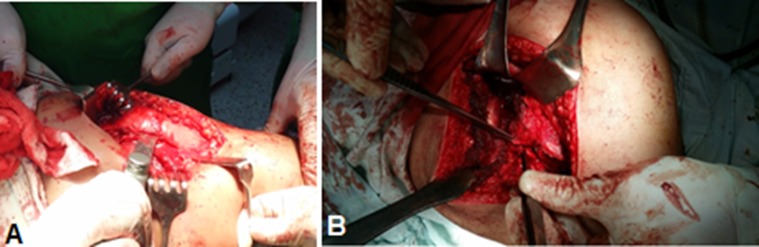
(A) Showing trochanteric osteotomy in patient No. 9. (B) Z-shaped capsulotomy in Lt hip in patient No. 11.

*A trigastric trochanteric osteotomy* (Figure 2A) was cut with an oscillating saw, leaving the trochanteric crest untouched. The 1 cm to 1.5 cm thick bony slice including the insertions of *gluteus medius and minimus with the insertion of vastus lateralis* was flipped anteriorly exposing the anterior hip capsule through the interval between the piriformis tendon and gluteus minimus.

*Dissection of the overlying anterior hip capsule* is continued as the greater trochanter is retracted further medially. After the entire anterior hip capsule has been exposed, a *Z-capsulotomy* (Figure 2B) is performed by first incising along the axis of the femoral neck and then extending proximally after the labrum is visualized and protected. The capsulotomy turns sharply at the acetabular rim and continues posteriorly in a curvilinear manner parallel to the labrum, back to the piriformis tendon. The capsulotomy is then opened to inspect and palpate the head and head-neck junction.

*Epiphyseal perfusion* is checked by inspecting the blood flow out of a simple drill hole from the periphery of the head directed toward the center, then division of the ligamentum teres to allow femoral head dislocation. The area where the retinacular vessels enter the epiphysis could be identified. The acetabulum was inspected for cartilage damage or labral tear.

*Development and release of the retinacular flap*, which composed of the periosteum of the femoral neck including the retinacular vessels, help to preserve the blood supply of the femoral head during the femoral head realignment. The periosteum was released approximately 4 cm distal to the greater trochanter.

*Further external rotation of the leg allowed thorough inspection* of the posteromedial part of the femoral neck and resection of the posterior buttress bone at this location. The femoral neck was shaped to allow tension-free repositioning of the femoral head centered above the neck.

*The remaining physis in the femoral head was curetted out* in order to accelerate bony healing after the femoral head was centered on the neck without tension on the retinaculum. Then provisional fixation with a K-wire and an intra-operative fluoroscopy was taken to ensure correct positioning was obtained, in particular the varus-valgus relationship.

**Figure 3.**
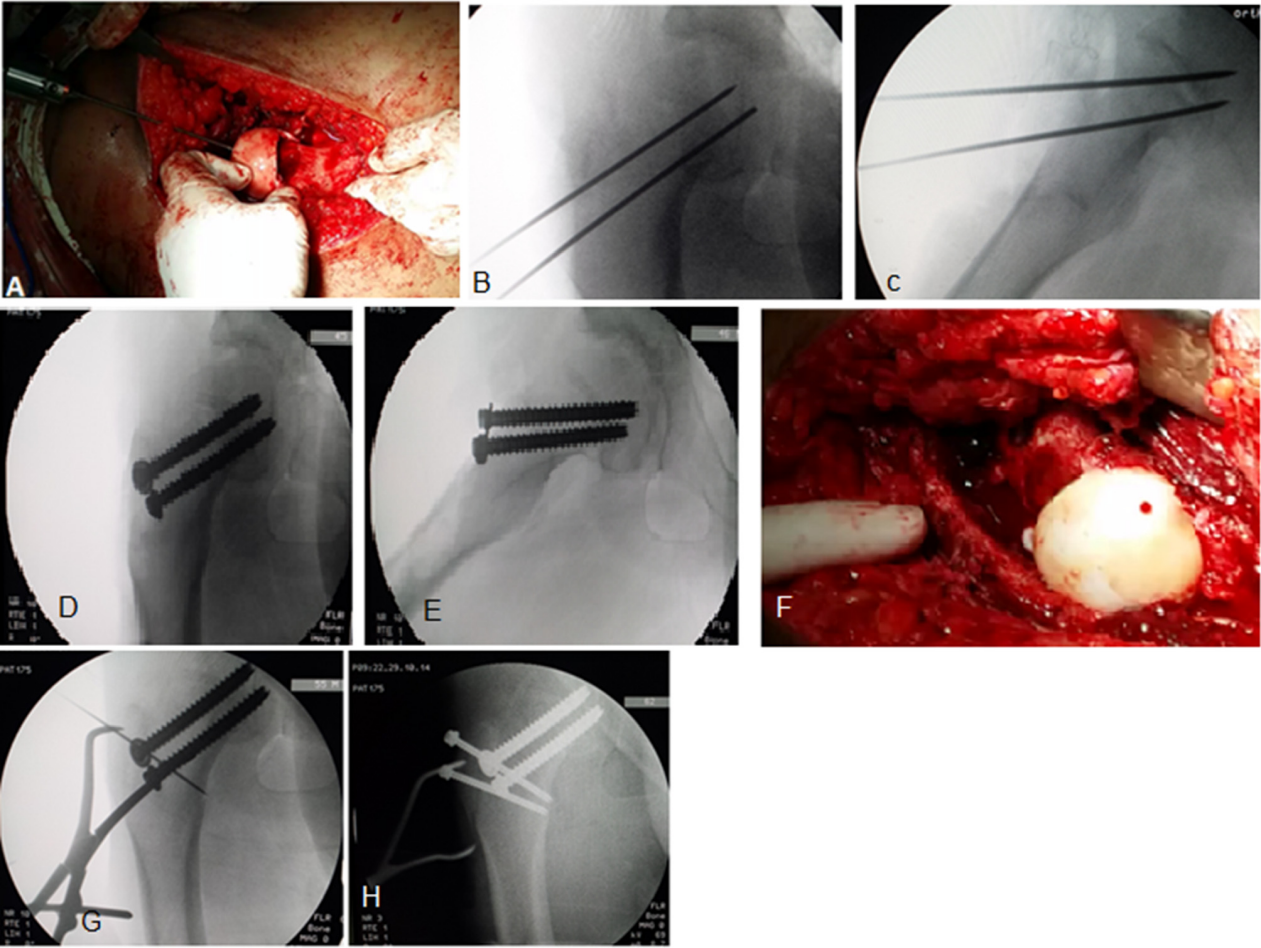
Intra-operative photograph (A) and the fluoroscopy images (B–E) were taken to ensure correct positioning and definitive fixation with two cancellous fully threaded screws, with bleeding head after definitive fixation (F), with reduction and fixation of the trochanteric osteotomy (G and H) in patient No. 9.

*The aim was to achieve* an anatomical position (Figures 3B and 3C) especially in relation to the fovea capitis and to avoid any varus malalignment as this would make the fixation less stable. The blood perfusion of the femoral head was rechecked, followed by definitive fixation with three fully threaded 3.0 mm K-wires or cannulated (6.5 mm) screws.

*The periosteal sleeve* and capsule were approximated with loose sutures and the greater trochanter was reattached with two cortical screws (4.5 mm) (Figures 3G and 3H). The fascia was accurately closed with a continuous suture followed by standard wound closure with a suction drain inside wound to prevent hematoma formation.

## Results

The mean operative time was (132.56 ± 29.5) minutes. Intraoperative blood loss was estimated by the anesthetics and it ranged from 100 cc to 500 cc with an average of 100 cc, none of our patients need intra-operative or postoperative blood transfusion. As regards the bleeding of head before dislocation as a test for viability the head was tested by peripheral drilling with K-wire, 30 head (93.75%) was positive bleeding before dislocation the remaining two heads (6.25%) with negative bleeding before dislocation. Bleeding of head after reduction was done to exclude tension on posterior retinaculum, 28 heads (87.5%) was positive bleeding after reduction, while the remaining four heads (12.5%) was negative bleeding after reduction, two of them (6.25%) were with negative bleeding before dislocation and two of them (6.25%) were with positive bleeding before dislocation.

As regards the method of fixation nine hips (30%) were fixed by cannulated 6.5 fully threaded screws and in 23 hips (70%) we used three fully threaded 3.0 mm K-wires. In our study, the mean postoperative slip angle was (5.6° ± 8.2°) with mean correction of (46.85° ± 14.9°). The mean postoperative alpha angle was (51.15° ± 4.2°) with mean correction of (46.7° ± 14.2°).

As regards postoperative range of motion (ROM) the mean postoperative flexion was (111.9° ± 19.05°). The mean postoperative IR in 90° flexion was (41.6° ± 9.6°). The mean postoperative ER in 90° flexion was (45.6° ± 9.97°).

As regards postoperative complication, in the majority of cases 84.3% no major postoperative complication occurred. One patient (3%) developed postoperative deep infection. Three cases (9.3%) developed postoperative AVN. One case (3%) with bad reduction needed revision.

We did not record any case of chondrolysis, OA, implant failure, symptomatic FAI, or heterotopic ossification (HO) occurred postoperatively.

As regards the mean postoperative HHS in our series it was (96.16 ± 9.7) and the mean improvement was (29.6 ± 9.6). The mean WOMAC score was (3.3 ± 3.5) and mean improvement was (85.12 ± 4.7). The mean Merle d’Aubigne score was (16.8 ± 2.4) and the mean improvement was (4.8 ± 2.4) and as regards the Hayman and Herndon score 30/32 hips were good and excellent results (Tables 2 and 3).

**Table 3.** Postoperative features of SCFE patients treated with modified Dunn procedure.

Patient number	LLD (cm)	Complication	Flexion	IR + flexion	ER + flexion	HHS score	WOMAC score	Merle score	Hayman score	Slip angle	Alpha angle
1	2	AVN	80	20	20	88	10	10	Good	0	55
2	2.5	AVN	70	10	20	66	11	10	Fair	0	50
3	0	Infection	120	45	50	100	0	18	Good	16	55
4	0	Negative	130	45	50	96	4	18	Good	1	45
5	0		100	45	50	100	3	18	Excellent	13	54
6	0		50	10	10	100	3	18	Good	2	55
7	2		100	40	45	68	12	11	Fair	−4	55
8	2		100	45	50	68	12	11	Good	0	55
9	0	AVN	130	45	50	100	0	18	Excellent	10	50
10		Negative	120	45	50	100	2	18	Good	9	55
11			120	45	50	100	4	18	Good	17	47
12			120	45	50	98	0	18	Excellent	0	50
13			100	40	50	100	0	18	Good	19	64
14			120	45	50	98	2	17	Excellent	3	45
15			100	45	45	100	0	18	Excellent	1	50
16			100	45	45	98	4	18	Good	−4.5	49.3
17			120	45	45	100	6	18	Excellent	2	55
18			100	45	45	100	0	17	Excellent	0	50
19			110	45	50	100	4	18	Excellent	28.3	54.3
20			110	45	45	100	0	18	Good	7	50
21			120	45	45	98	3	17	Excellent	11	55
22			130	45	50	100	0	17	Excellent	6.8	53.9
23			130	45	50	100	0	18	Excellent	5	50
24			120	45	50	99	2	18	Good	11	47.8
25			130	45	50	100	4	18	Excellent	−12.2	45.5
26			120	45	50	100	4	18	Good	0	50
27			130	45	50	100	4	16	Excellent	18	48
28			130	45	50	100	0	18	Good	2	45
29			120	45	50	100	4	18	Excellent	0	50
30			110	45	50	100	4	18	Excellent	10	54
31			130	45	50	100	0	16	Good	4	47
32			80	20	20	100	5	18	Excellent	8	47

## Discussion

Our results show that repositioning the epiphysis and restoration of normal proximal femoral anatomy in SCFE are possible with a low risk of AVN by reducing the tension on the posterosuperior retinaculum which contains the end branches of the medial femoral circumflex artery by wedge resection of posterior callus formed due to slippage.

*According to Southwick radiological classification* our study is the largest study including those with severe SCFE we had 20 patients who were classified as severe, 10 patients as moderate, and two patients as mild. In Ziebarth et al. [22] study, 23 patients were classified as moderate and 12 patients were severe, in Huber et al. [25] study, three patients were classified as mild, 17 were moderate, and 10 patients were severe, and in Slongo et al. [24] study, six patients were classified as mild, eight were moderate, and nine patients were severe. In Novais et al. [26] study, 15 patients (100%) were severe. In Cosma et al. [27] study, seven patients (100%) were severe (Table 4).

**Table 4.** Comparison of patient criteria in different studies.

		Ziebarth et al. (2009)	Slongo et al. (2010)	Huber et al. (2011)	Masse et al. (2012)	Sankar et al. (2013)	Novais et al. (2015)	Cosma et al. (2016)	Current study
No. of pts.		40 (40 hips)	23 (23 hips)	28 (30 hips)	19 (20 hips)	27 (27 hips)	15 (15 hips)	7 (7 hips)	30 (32 hips)
Age at operation	Range	16-Sep.	17-Jul.	9.4–16.6	19-Sep.	9.7–16.0	17-Dec.	12–13	18-Oct.
	Mean	12.5	12	12.2	14.2	12.6	14	13	14
Sex	Male	17 (42.5%)	14 (60.9%)	11 (39.3%)	–	17 (63%)	11 (73%)	7 (100%)	22 (73.3%)
	Female	23 (57.5%)	9 (39.1%)	17 (60.7%)	–	10 (37%)	4 (27%)	0	8 (26.7%)
Side	Rt	12 (30%)	9 (39.13%)	–	–	9 (33%)	9 (60%)	3 (42.9%)	17 (56.7%)
	Lt	28 (70%)	14 (60.8%)	–	–	18 (66.6%)	6 (40%)	4 (57.1%)	11 (36.7%)
	Bilateral				–				2 (6.6%)
Duration of symptoms	Range	1 day–3 years	2 days–224 day	–	–	6–184 h)	–	3.5–5.5 weeks	1–10 months
	Mean	4 months	35 day	–	–	35.9 h	–	5 weeks	4.5 months
Follow-up (months)	Range		23–62	15–102	24-Jun.	Dec.-48	12–60	8.5–23	Jun.-36
	Mean	42	24	51.6	10.65	22.3	28.8	12	17.3
Fahey classification	**Acute**	**13 (32.5%)**	**–**	**3 (10%)**	**2 (10%)**	**27 (100%)**	**0**	**2 (28.6%)**	**0**
	Acute on chronic	0	14 (60.9%)	0		0		1 (14.3)	1 (3%)
	Chronic	27 (67.5%)	9 (39.1%)	27 (90%)	18 (90%)	0	15 (100%)	4 (57%)	31 (97%)
Loder classification	Stable	27 (67.5%)	20 (87%)	27 (90%)	18 (90%)	0	15 (100%)	6 (85.7%)	32 (100%)
	Unstable	13 (32.5%)	3 (13%)	3 (10%)	2 (10%)	27 (100%)	–	1 (14.3%)	–
Southwick classification	Mild	5 (12.5%)	6 (23%)	3 (10%)		–	–	0	2 (6.25%)
	Moderate	23 (57.5%)	8 (35%)	17 (57%)		–	–	0	10 (31.25%)
	Severe	12 (30%)	9 (42%)	10 (33%)	20 (100%)	–	15 (100%)	7 (100%)	20 (62.5%)
Pre op Slip angle	**Range**	**34–70**°	**39–57**°	**19–77**°		**–**	**54–81**°	**64–71.5**°	**23–82.1**°
	Mean	45.6	47.6	44.9	50.65	–	650	680	52.5
Postoperative slip angle	Range	1° to 20°	3.5° to 6°	−430		2 to 11°	6 to 23°	7.5 to 13.5°	−12.2 to 28°
	Mean	8.6	4.6	5.2	9.45	60	160	90	5.6
	Mean correction	370	430	39.7	–	–	–		46.85
Pre op alpha angle	Range	–	–	–	–	–	93–120°	–	75–146.3°
	Mean				–	–	1110	–	97.85
Postoperative alpha angle	Range	27°–60°	24°–53°	27°–77°	–	–	40–51°	–	45–64°
	Mean	40.6	380	41.4	43.11	–	440	–	51.15°
	Mean correction	–	–	–		–		–	46.7

*In our study preoperative slip angle* ranged from 23° to 82.1° with mean of 52.5°. Postoperative slip ranged from −12.2° to 28° with mean of 5.6°, mean correction of 46.85°. In Ziebarth et al. study [22] preoperative slip angle ranged from 34° to 70° with mean of 45.6°, postoperative slip angle ranged from 1° to 20° with mean of 8.6°, mean correction of 37°. In Huber et al. [25] study preoperative slip angle ranged from 19° to 77° with mean of 44.9°, postoperative slip angle ranged from −18° to 25° with mean of 5.2°, mean correction of 39.7°. In Slongo et al. [24] study preoperative slip angle ranged from 39° to 57° with mean of 47.6°, postoperative slip angle ranged from 3.5° to 6° with mean of 4.6°, mean correction of 43°. In Novais et al. [26] study preoperative slip angle ranged from 54° to 81° with mean of 65°, postoperative slip angle ranged from 6° to 23° with mean of 16°. In Cosma et al. [27] preoperative slip angle ranged from 64° to 71.5° with mean of 68°, postoperative slip angle ranged from 7.5° to 13.5° with mean of 9°. This reveals that our mean correction of slip angle is the highest due to the inclusion of more severe cases (Table 4).

*In our study, postoperative alpha angle* was restored to normal. It ranged from 45° to 64° with mean of 51.15°, with mean correction of 46.7°. In Ziebarth et al. [22] study postoperative alpha angle ranged from 27° to 60° with mean of 40.6°, in Huber et al. [25] study postoperative alpha angle ranged from 27° to 77° with mean of 41.4°, in Slongo et al. [24] study postoperative alpha angle ranged from 24° to 53° with mean of 38°. In Novais et al. [26] study postoperative alpha angle ranged from 40° to 51° with mean of 44° (Table 4).

*In comparison to other studies we restored near-normal postoperative ROM*. In our study postoperative flexion ranged from 50 to 130 with mean of 111.9, in Ziebarth et al. [22] study postoperative flexion ranged from 80 to 120 with mean of 104, in Huber et al. [25] study postoperative flexion was more than 90° except in a patient who developed AVN, in Slongo et al. [24] study postoperative flexion ranged from 20 to 130 with mean of 107.

Postoperative IR in 90° flexion ranged from 10 to 45 with mean of 41.6, in Ziebarth et al. [22] study postoperative IR in 90° flexion ranged from 5 to 45 with mean of 29, in Huber et al. [25] study postoperative IR in 90° flexion ranged from 10 to 50 with mean of 33.3, in Slongo et al. [24] study postoperative IR in 90° flexion ranged from 10 to 60 with mean of 37.8.

Postoperative ER in 90° flexion ranged from 15 to 50 with mean of 45.6, Ziebarth et al. [22] study postoperative ER in 90° flexion ranged from 20 to 60 with mean of 43, in Huber et al. [25] study postoperative ER in 90° flexion ranged from 20 to 70 with mean of 49.8, in Slongo et al. [24] study postoperative ER in 90° flexion was ranged from 10 to 60 with mean of 45.

*We evaluated the postoperative clinical outcome* by use of four different clinical scores. This allowed us to compare our results with the results of other published studies. The HHS in our series ranged from 66 to 100 with mean of 96.16. The mean postoperative HHS in Ziebarth et al. [22] study was 99.6, in Huber et al. [25] study postoperative HHS ranged from 56 to 100 with mean of 97.8, and in Slongo et al. [24] study postoperative HHS ranged from 82 to 100 with mean of 99. In comparison to other studies our mean HHS was slightly lower due to involvement of more chronic cases which had slight persistent postoperative pain and limited ROM due to muscle weakness. This may improve on long-term follow-up with return of the patients to their full activity and regaining full muscle power.

The WOMAC score ranged from 0 to 12 with mean of 3.3 while in Massè et al. [28] study, postoperative pain score at WOMAC was 0.6 (ranged from 0 to 4) and postoperative function score at WOMAC was 2, 2 (ranged from 0 to 12). The Merle d’Aubigne score ranged from 10 to 18 with mean of 16.8. In Ziebarth et al. [22] study the mean Merle d’Aubigne score was 17.8, in Slongo et al. [24] study the Merle d’Aubigne score ranged from 11 to 18 with mean of 17. The Heyman and Herndon score in our series was 30/32 had good and excellent results. In Novais et al. [26] study the Heyman and Herndon score was 9/15 had good and excellent results. In Cosma et al. [27] study the Heyman and Herndon score was 6/7 had good and excellent results (Table 5).

**Table 5.** Comparison of postoperative clinical scores in different studies.

Postoperative clinical scores		Ziebarth et al. (2009)	Slongo et al. (2010)	Huber et al. (2011)	Masse et al. (2012)	Sankar et al. (2013)		Novais et al. (2015)	Cosma et al. (2016)	Current study
						No AVN	AVN			
HHS	Range		82–100	56–100		86.7–89.3	44.8–75.1			66–100
	Mean	99.6	99	97.1	98.2	88.0	60.0			96.16
WOMAC	Range	–	–	–	Pain (0–2) Function (0–12)	–	–			0–12
	Mean	Pain (2.1) Function (3)	–	–	Pain (0.6) Function (2.2)	–	–			3.3
Merle d’Aubigne	Range		11–18	–		–	–			10–18
	Mean	17.8	17	–		–	–			16.8
Heyman and Herndorn								9/15 had good and excellent results	6/7 had good and excellent results	30/32 had good and excellent results

*As regards the modified Dunn osteotomy* with surgical hip dislocation, the incidence of complications was low. In our study three cases (9.3%) developed postoperative AVN. In Huber et al. [25] study they recorded one case of AVN (3.5%), in Massè et al. [28] study they did not record any case of AVN, in Slongo et al. [24] study they also recorded one case of AVN (4.4%), Sankar et al. [29] study recorded seven cases of AVN (26%), Novais et al. [26] study recorded one case of AVN (7%), Cosma et al. [27] did not record any case of AVN.

We did not record any case with implant failure. In Huber et al. [25] study they recorded four cases of implant failure necessitating revision of fixation with cortical screws. While in Ziebarth et al. [22] study they recorded three cases of implant failure which necessitated revision of surgery, Slongo et al. [24] study recorded one case of implant protrusion that needed revision of fixation. Sankar et al. [29] recorded four cases of implant failure which necessitate revision of surgery. Novais et al. [26] recorded two cases of implant failure which necessitate revision of surgery.

In our series, the need for reoperation was low in comparison to other series. Only three patients needed reoperation; one of the three who developed postoperative AVN needed another surgery to remove the protruded screws and arthrodiastasis, one with late deep infection needed debridement and screw removal, one with bad reduction needed revision with adjustment of the reduction. Most of the reoperations in other studies were due to revision of failed fixation which did not occur in our study. Another cause of reoperation was development of postoperative HO which developed after correction of acute slippage but in our study all cases were chronic so avoiding these complications. Sankar et al. [29] recorded one patient (3.7%) who needed open osteoplasty of residual deformity due to AVN, one patient (3.7%) of AVN who needed core decompression, and another patient of AVN (3.7%) who needed total hip replacement (THR) in addition to revision of failed implants and removal of protruded implant in nine patients (33.5%) (Table 6).

**Table 6.** Comparison of the incidence of postoperative complications in different studies.

	The Dunn osteotomy	Modified Dunn osteotomy							
	Lawane et al. (2009)	Ziebarth et al. (2009)	Slongo et al. (2010)	Huber et al. (2011)	Masse et al. (2012)	Sankar et al. (2013)	Novais et al. (2015)	Cosma et al. (2016)	Our study
Infection	0	0	0	0	0	0	0	0	1 (3%)
Implant failure	0	3 (7.5%)	1 (4.4%)	4 (13.5%)	0	4 (15%)	2 (13%)	0	0
H.O	0	3 (7.5%)	0	0	0	0	0	0	0
Delayed union	1 (4%)	3 (7.5%)	0	0	0	0	0	0	0
AVN	5 (20%)	0	1 (4.4%)	1 (3.5%)	0	7 (26%)	1 (7%)	0	3 (9.4%)
Chondrolysis	3 (12%)	–	–	–	0	–		0	0
Limited ROM		0	1 (4.4%)	0	0	7 (26%) due to AVN	6 (40%)	1 (14.2%)	3 (9.4%) due to AVN
OA		0	1 (4.4%)	0	0		1 (7%)	0	0
FAI	1 (4%)	1 (2.5%)	0	0	0	1 (3.7%)	0	0	0
Total incidence of complications	10 (40%)	10 (25%)	4 (17.6%)	5 (17%)	0	11 (41%)	3 (20 %)	1 (14.2%)	5 (15.6%)

### The strength and limitations

*The strong points* in the current study can be summarized in three points. The first point is that our study is the largest study dealing with management of chronic stable SCFE using *Ganz surgical hip dislocation*. The second point is that it is the only study that uses four different clinical scores to assess postoperative clinical outcome and this allows feasibility to compare our results with the results of other published studies. The third point is that we had the largest mean correction of slip angle postoperatively probably due to inclusion of more severe cases.

*On the other side, this case series study has some major limitations*, like any single cohort study; there was lack of comparison or control group in this case series study. Without a truly randomized long-term study about results of *Ganz* surgical hip dislocation it would be difficult to compare outcomes of this technique with those of various other surgical techniques such as in situ pinning, capital realignment, and intertrochanteric osteotomy. Moreover, it was done by two different surgeons in two hospitals which had a direct influence on the outcome and postoperative results. Another major limitation in our study is lack of long-term follow-up in comparison with other studies. This was due to late application of this technique in our institution in 2013, while in other studies they started very early in 1996 [23], 1998 [22], 2001 [25], and 2004 [24]. One of the major limitations was our dependence on rough method to detect head viability (peripheral K-wire drilling) which lacks both sensitivity and specificity. The use of Doppler ultrasound probe can detect perfusion of both epiphysis and retinaculum so can be a good reliable test for predicting the development of postoperative AVN.

## Case presentation

Figures 4 and 5.

**Figure 4.**
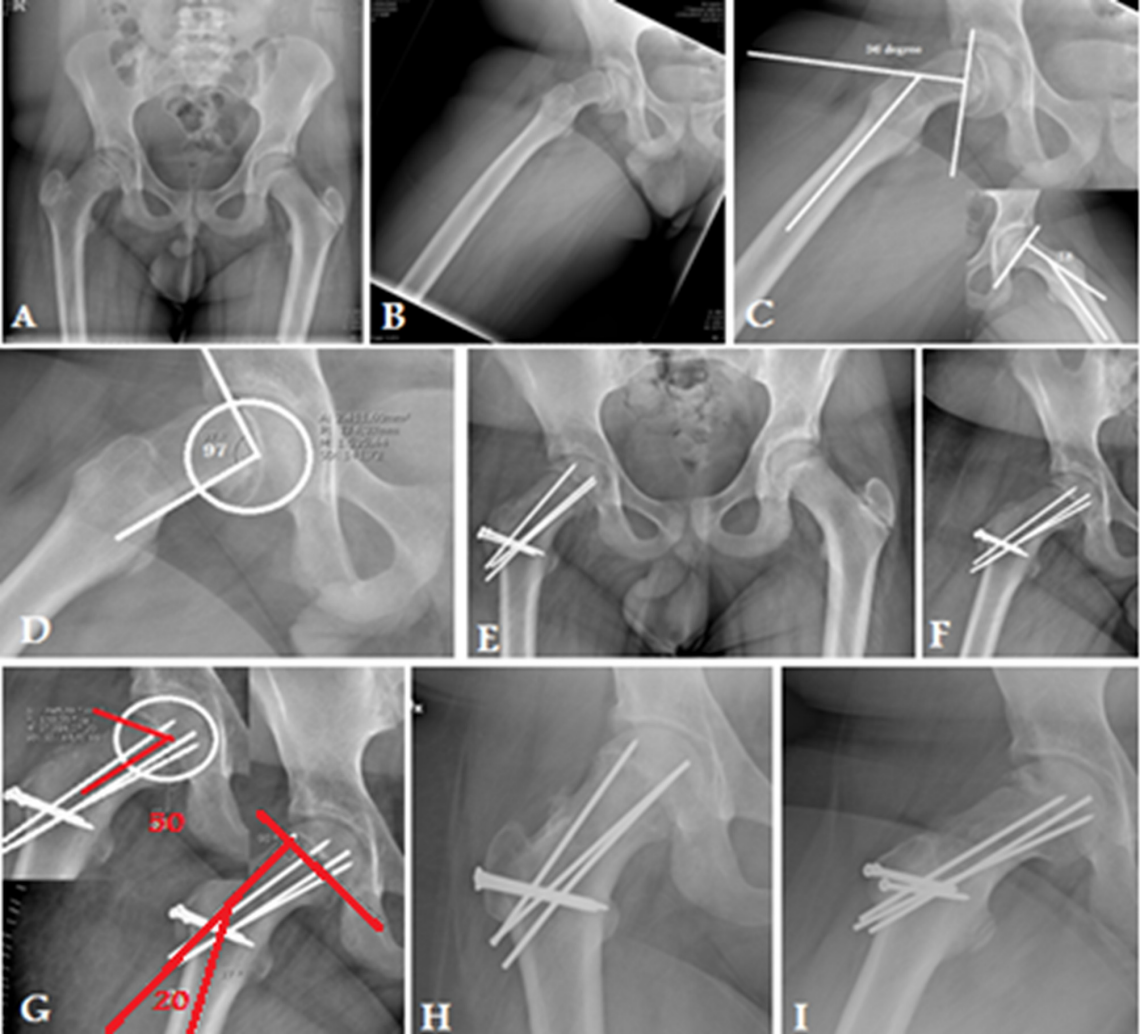
X-ray in a 15-year-old male with Rt chronic stable SCFE (case number 10): (A) preoperative AP pelvis, (B) preoperative frog lateral view of the Rt hip, (C) preoperative measurement of Southwick slip angle, (D) preoperative measurement of alpha angle, (E) and (F) postoperative anteroposterior (AP) and frog view, (G) postoperative measurement of Southwick slip and alpha angles, (H) 16-month postoperative AP and frog lateral X-ray showing complete union of trochanteric osteotomy and physis with no evidence of AVN.

**Figure 5.**
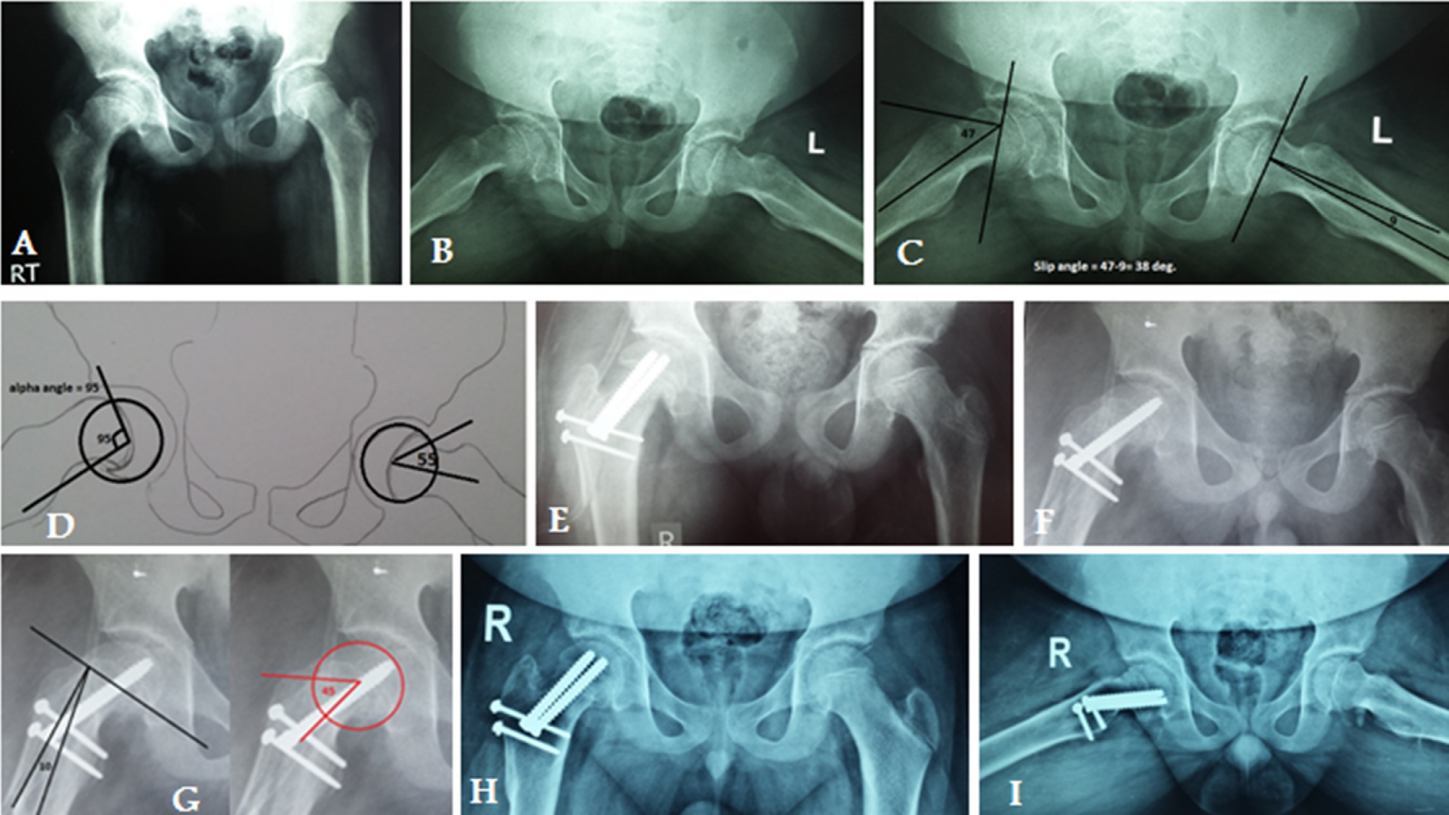
X-ray in 15-year-old male with Rt chronic stable SCFE (case number 4): (A) preoperative AP pelvis, (B) preoperative frog lateral view of both hips, (C) preoperative measurement of Southwick slip angle, (D) preoperative measurement of the alpha angle, (E) and (F) postoperative AP and frog view, (G) postoperative measurement of Southwick slip and alpha angles, (H) 12-month postoperative AP and frog lateral X-ray showing complete union of trochanteric osteotomy and physis with no evidence of AVN.

## Conclusion

*The modified Dunn osteotomy using Ganz surgical hip dislocation* is our treatment of choice for those with moderate and severe SCFE, allowing anatomical restoration of proximal femur, direct inspection, and preservation of physeal blood supply and inspection of intra-articular pathology which can be evaluated and treated at the time of operation. In the majority of cases we can relocate the epiphysis without need for shortening the femoral neck. The modified Dunn procedure is safe, efficient, and reproducible, but it has a long learning curve and it should be learned in a specialized center before using it in clinical practice.

## Conflict of interest

The authors declare no conflict of interest in relation with this paper.
